# Entropy Interpretation of Hadamard Type Fractional Operators: Fractional Cumulative Entropy

**DOI:** 10.3390/e24121852

**Published:** 2022-12-19

**Authors:** Vasily E. Tarasov

**Affiliations:** 1 Skobeltsyn Institute of Nuclear Physics, Lomonosov Moscow State University, Moscow 119991, Russia; tarasov@theory.sinp.msu.ru; 2 Department of Physics, 915, Moscow Aviation Institute (National Research University), Moscow 125993, Russia

**Keywords:** fractional calculus, fractional integrals, Hadamard-type fractional pperator, entropy, cummulative entropy, fractional entropy, 26A33, 94A17

## Abstract

Interpretations of Hadamard-type fractional integral and differential operators are proposed. The Hadamard-type fractional integrals of function with respect to another function are interpreted as an generalization of standard entropy, fractional entropies and cumulative entropies. A family of fractional cumulative entropies is proposed by using the Hadamard-type fractional operators.

## 1. Introduction

Integrals and derivatives of arbitrary (non-integer and integer) orders form a generalization of standard calculus of integrals and derivatives of integer orders (see books [1,2,3,4,5] and handbooks [6,7]). These operators were proposed by famous mathematicians such as Riemann, Liouville, Grünwald, Letnikov, Sonine, Marchaud, Weyl, Riesz, Hadamard and others [8,9,10]. Fractional integrals and derivatives have a wide application in physics and mechanics to describe processes and systems with non-locality, memory, distributed lag and scaling [11,12,13], various anomalous behaviors and phenomena, including the relaxation–oscillation phenomena, [14,15], the diffusion-wave phenomena [14,16], the anomalous relaxation [17,18,19] and anomalous growth [19,20], and the anomalous diffusion and anomalous transport [21,22,23,24,25].

There are various interpretations of the fractional integrals and derivatives [26,27,28,29,30,31,32,33,34] such as geometric interpretations [35,36,37,38] and [39,40,41,42], physical interpretations [39,40,41,42] and [30,31,32,33,34], economic interpretations [43,44], probabilistic interpretations [45,46,47,48,49], information interpretation [50] and some others.

The so-called Hadamard fractional integrals were proposed by Jacques S. Hadamard [51] in 1892. These operators and their properties are described in [4] (pp. 110–120), (see also Section 18.3, 23.1 of [1] and [52]). The Hadamard-type fractional integral (HTFI) and fractional differential (HTFD) operators were first suggested by Paul L. Butzer, Anatoly A. Kilbas and Juan J. Trujillo in 2002 [53,54]. These operators can be called the Butzer–Kilbas–Trujillo operators. The properties of the HTFD and HTFI operators are described in papers [53,54,55,56,57,58,59]. Caputo-type modifications of the Hadamard fractional derivatives are considered in [60,61,62]. The Hadamard fractional calculus is also considered in [63,64,65]. The application of fractional differential equations with Hadamard operators in probability theory is proposed in article [64,66]. The HTFI and HTFD operators to describe dynamics with non-local scaling is suggested in [67]. The Hadamard-type fractional integrals and derivatives with respect to functions are proposed in work [68] in 2021. If the function is equal to the integration variable Ψ(x)=x, then these operators have the form of the HTFI and HTFD operators.

In the proposed paper, an entropy interpretation of Hadamard-type fractional integrals with respect to functions is proposed. It will be shown that these operators can be interpreted as generalized cumulative entropy. In Section 2, the Hadamard-type fractional integral and differential operators are defined. In Section 3, cumulative and fractional entropies as generalizations of standard continuous (differential) entropy are discussed. In Section 4, entropy interpretation of the Hadamard-type fractional integral and differential operators is considered. A brief conclusion is given in Section 5.

## 2. Hadamard-Type Fractional Operators

### 2.1. Hadamard-Type Fractional Integrals

Let us give a definition of space that is used for the Hadamard-type fractional integrals.

**Definition** **1.** *The space $X_{q}^{p}\left(a,b\right)$ with q∈R and p≥1 is the weighted $L_{p}$-space with the power weight, which consists of those complex-valued Lebesgue measurable functions X(t) on (a,b) for which*

(1) $${\left||X|\right|}_{X_{q}^{p}}={\left(\int_{a}^{b}\frac{d\tau }{\tau }{\left|\tau^{q}X\left(\tau \right)\right|}^{p}\right)}^{1/p}<\infty .$$

*In particular, when q=1/p, the space $X_{q}^{p}\left(a,b\right)$ coincides with the space $L_{p}\left(a,b\right)$, i.e., $X_{1/p}^{p}\left(a,b\right)=L_{p}\left(a,b\right)$.*

The HTFI operator of the order α>0 is given by the following definition [53,54].

**Definition** **2.** *The Hadamard-type fractional integral (HTFI) operator of the order α>0 is defined by the expression*

(2) $$J_{a,x}^{\alpha ,\beta }\left[t\right]f\left(t\right)\;=\;\frac{1}{\Gamma (\alpha )}\int_{a}^{x}\frac{dt}{t}\;{\left(\frac{t}{x}\right)}^{\beta }{\left(\ln\frac{x}{t}\right)}^{\alpha -1}\;f\left(t\right),$$

*where β∈R, x∈(a,b), a>0.*

The operator $J_{a,x}^{\alpha ,\beta }$ is bounded in the space $X_{q}^{p}\left(a,b\right)$, where β∈R, β>q, p≥1, a>0 (see Theorem 2.1 in [54] (p.1194)).

For α=m∈N, operator (2) has [54] (p. 1191) the form

$$J_{a,t}^{m,\beta }\left[t\right]f\left(t\right)\;=\;x^{-\beta }\int_{a}^{x}\frac{dx_{1}}{x_{1}}\int_{a}^{x_{1}}\frac{dx_{2}}{x_{2}}\;.\;.\;.\int_{a}^{x_{m-1}}t^{\beta }\;f\left(t\right)\frac{dt}{t}\;=\;$$

(3) $$\frac{1}{(m-1)!}\int_{a}^{x}{\left(\frac{t}{x}\right)}^{\beta }\;{\left(\ln\frac{x}{t}\right)}^{m-1}\;f\left(t\right)\;\frac{dt}{t}.$$

Integral operator (2) with β=0 is called the Hadamard FI, which was proposed by Jacques S. Hadamard [51] in 1892. These operators and their properties are described in [4], pp. 110–120 (see also Sections 18.3, 23.1 of [1,52]).

### 2.2. Hadamard-Type Fractional Differential Operator

Let us give a definition of space $AC_{S,\beta }^{m}\left[a,b\right]$ that is used for the Hadamard-type fractional differential operators [54] (p.1193).

**Definition** **3.** *The space $AC_{S,\beta }^{m}\left[a,b\right]$ consists of functions f(x) on [a,b] that have $S_{x}^{k}\left(x^{\beta }\;f\left(x\right)\right)$ for k=1,...m−1, and $S_{x}^{m-1}\left(x^{\beta }\;f\left(x\right)\right)$ is absolutely continuous on [a,b], where $S_{x}=x\;d/dx$.*

Let us define the differential operator of integer order m∈N in the form

(4) $$D_{x}^{m,\beta }\;f\left(x\right)\;=\;x^{-\beta }S_{x}^{m}\;\left(x^{\beta }\;f\left(x\right)\right)\;=\;x^{-\beta }{\left(x\frac{d}{dx}\right)}^{m}\;\left(x^{\beta }\;f\left(x\right)\right).$$

Let us give the definition of the HTFD operator (for example, see Section 18.3 in [1,4] (pp. 11–112)).

**Definition** **4.** *The Hadamard-type fractional differential (HTFD) operator of the order α∈[m−1,m), m∈N is defined by the expression*

(5) $$D_{a,x}^{\alpha ,\beta }\left[t\right]\;f\left(t\right)\;=\;D_{x}^{m,\beta }\;J_{a,x}^{m-\alpha ,\beta }\left[t\right]\;f\left(t\right)\;=\;$$

$$\frac{1}{\Gamma (m-\alpha )}x^{-\beta }\;{\left(x\frac{d}{dx}\right)}^{m}x^{\beta }\int_{a}^{x}\frac{dt}{t}{\left(\frac{t}{x}\right)}^{\beta }{\left(\ln\frac{x}{t}\right)}^{m-\alpha +1}\;f\left(t\right),$$

*where $J_{a,x}^{m-\alpha ,\beta }$ is the HTFI operator, x>a>0.*

The operator $D_{a,x}^{\alpha ,\beta }$ exists almost everywhere on the space $AC_{S,\beta }^{m}\left(a,b\right)$. This statement is proved as Theorem 3.2 in [54] (p. 1198).

For α=m∈N, the HTFD operator is the integer-order differential operator [4] (p. 112), in the form

(6) $$D_{a,x}^{m,\beta }\;f\left(x\right)\;=\;D_{x}^{m,\beta }\;f\left(x\right).$$

Operator (5) with β=0 is called the Hadamard fractional differential operators [4] (pp. 110–120), and [52]. For β=0, and α=m∈N, the HTFD operator is the differential operator of scaling (dilation) of the integer order

(7) $$D_{a,x}^{m,0}\;f\left(x\right)\;=\;S_{x}^{m}\;f\left(x\right)\;=\;{\left(x\frac{d}{dx}\right)}^{m}\;f\left(x\right).$$

**Definition** **5.** *The Caputo-type of the HTFD operator of the order m−1≤α<m is defined as*

(8) $${}^{C}D_{a,x}^{\alpha ,\beta }\left[t\right]\;f\left(t\right)\;=\;J_{a,x}^{m-\alpha ,\beta }\left[t\right]D_{t}^{m,\beta }\;f\left(t\right)\;=\;$$

$$\frac{1}{\Gamma (m-\alpha )}\int_{a}^{x}\frac{dt}{t}{\left(\frac{t}{x}\right)}^{\beta }{\left(\ln\frac{x}{t}\right)}^{m-\alpha +1}\;\left(t^{-\beta }{\left(t\frac{d}{dt}\right)}^{m}t^{\beta }\right)x\;f\left(t\right),$$

*where $J_{a,x}^{m-\alpha ,\beta }$ is the HTFI operator, x>a>0.*

### 2.3. Hadamard-Type FI and FD Operators with Respect to Function

Let us give a definition of space that is used for the Hadamard-type fractional integrals with respect to function. This definition gives conditions under which the Hadamard-type fractional integral operator $J_{\Psi ,a,x}^{\alpha ,\beta }\left[t\right]$ with respect to a function Ψ(x) is bounded in a particular function space $X_{\Psi ,q}^{p}\left(a,b\right)$. The space $X_{\Psi ,q}^{p}\left(a,b\right)$ is defined in [68] following [53,54] for the Hadamard-type fractional calculus.

**Definition** **6.** *Let q∈R and 1≤p≤∞. The space $X_{\Psi ,q}^{p}\left(a,b\right)$ is defined to consist of all Lebesgue measurable functions f:[a,b]→R, for which ${∥f∥}_{X_{\Psi ,q}^{p}}<\infty$, where the norm is defined by*

(9) $${\left||f|\right|}_{X_{\Psi ,q}^{p}}\;=\;{\left(\int_{a}^{b}{\left|{\left\{\Psi (x)\right\}}^{q}f\left(x\right)\right|}^{p}\frac{\Psi^{\prime }\left(x\right)}{\Psi (x)}\;dx\right)}^{\frac{1}{p}}\;for\;q\;\in \;R,\;1\;\leq \;p\;<\;\infty ,$$

*and*

(10) $${\left||f|\right|}_{X_{\Psi ,q}^{\infty }}\;=\;\underset{x\in [a,b]}{\sup}\left({\left\{\Psi (x)\right\}}^{q}\left|f(x)\right|\right),\;for\;q\;\in \;R.$$

If we consider q=1/p and Ψ(x)=x, then the space $X_{\Psi ,q}^{p}\left(a,b\right)$ coincides with the space $L^{p}\left(a,b\right)$ with

$${∥f∥}_{p}\;=\;{\left(\int_{a}^{b}{\left|f(x)\right|}^{p}dx\right)}^{\frac{1}{p}}\;for\;1\;\leq p\;<\;\infty ,$$

and

$${∥f∥}_{\infty }\;=\;\underset{x\in [a.b]}{\sup}\left|f(x)\right|.$$

Let us give a definition of the HTFI with respect to function.

**Definition** **7.** *Let α>0, and β∈R. Let f(x) be an integrable function defined on [a,b] in R and $\Psi \left(x\right)\in C^{1}\left(\left[a,b\right]\right)$ be a positive increasing function such that Ψ(x)>0 and $\Psi^{\left(1\right)}\left(x\right)=d\Psi \left(x\right)/dx>0$ for all x∈[a,b].*

*Then, the Hadamard-type fractional integral of f(x) with respect to Ψ(x) with order α and parameter β is defined as*

(11) $$J_{\Psi ,a,x}^{\alpha ,\beta }\left[t\right]f\left(t\right)\;=\;\frac{1}{\Gamma (\alpha )}\int_{a}^{x}{\left(\frac{\Psi (t)}{\Psi (x)}\right)}^{\beta }{\left(\ln\frac{\Psi (x)}{\Psi (t)}\right)}^{\alpha -1}\;f\left(t\right)\;\frac{\Psi^{\left(1\right)}\left(t\right)}{\Psi (t)}\;dt,$$

*where $f\left(x\right)\in X_{\Psi ,q}^{p}\left(a,b\right)$ and β>q.*

In paper [68], Theorem 4.2 states that for a positive increasing function Ψ(x) and for any β≥q, the Hadamard-type fractional integral operator of any positive order α>0 and parameter β≥q with respect to Ψ(x) is well-defined on the space $X_{\Psi ,q}^{p}\left(a,b\right)$.

**Theorem** **1.** *Let α>0, 1≤p≤∞, and β≥q in R, and Ψ(x) be a positive increasing function.*

*Then, the operator $J_{\Psi ,a,x}^{\alpha ,\beta }\left[t\right]$ is bounded in the space $X_{\Psi ,q}^{p}\left(a,b\right)$ with β≥q, and*

(12) $${∥J_{\Psi ,a,x}^{\alpha ,\beta }\left[t\right]f\left(t\right)∥}_{X_{\Psi ,q}^{p}}\;\leq C\;{∥f∥}_{X_{\Psi ,q}^{p}},$$

*where the constant C is defined by*

(13) $$C\;=\;\left\{\begin{array}{cc} \frac{1}{\Gamma (\alpha +1)}{\left(\ln\frac{\Psi (b)}{\Psi (a)}\right)}^{\alpha }\; & for\;\beta \;=\;q,\\ \frac{1}{\Gamma (\alpha )}{\left(\beta -q\right)}^{-\alpha }\gamma \left(\alpha ,\left(\beta -q\right)\ln\frac{\Psi (b)}{\Psi (a)}\right)\; & for\;\beta \;>\;q, \end{array}\right.$$

*where γ(α,z) is the incomplete gamma function (see Section 9 in [69] (pp. 134–142)).*

This theorem was proved in paper [68].

Let us define the Hadamard-type fractional differential operator of f(x) with respect to another function Ψ(x).

**Definition** **8.** *Let n=[α]+1, where n−1≤α<n. The Riemann–Liouville type of the Hadamard-type fractional differential operator of f(x) with respect to Ψ(x) of the order α>0 and parameter β∈R, is defined by the equation*

(14) $$D_{\Psi ,a,x}^{\alpha ,\beta }\left[t\right]\;f\left(t\right)\;=\;D_{\Psi ,x}^{n,\beta }\;J_{\Psi ,a,x}^{n-\alpha ,\beta }\left[t\right]\;f\left(t\right),$$

*where x∈[a,b]. The Caputo type of HTFD with respect to Ψ(x) is defined as*

(15) $$D_{\Psi ,a,x}^{\alpha ,\beta }\left[t\right]\;f\left(t\right)\;=\;J_{\Psi ,a,x}^{n-\alpha ,\beta }\left[t\right]\;D_{\Psi ,t}^{n,\beta }\;f\left(t\right),$$

*where $D_{\Psi }^{n,\beta }$ is the derivative of the integer order n∈N in the form*

(16) $$D_{\Psi ,x}^{n,\beta }\;=\;{\left(\Psi \left(x\right)\right)}^{-\beta }{\left(\frac{\Psi (x)}{\Psi^{\left(1\right)}\left(x\right)}\;\frac{d}{dx}\right)}^{n}\left({\left(\Psi \left(x\right)\right)}^{\beta }\;f\left(x\right)\right).$$

To give sufficient conditions for the existence of the HTFD operator $D_{\Psi ,a,x}^{\alpha ,\beta }$ with respect to a function Ψ(x), the function space $AC_{\delta^{\Psi },\beta }^{n}\left[a,b\right]$ for β∈R, and Ψ(x), which is a positive increasing function, should be defined. The space $AC_{\delta^{\Psi },\beta }^{n}\left[a,b\right]$ is defined as

(17) $$AC_{\delta^{\Psi },\beta }^{n}\left[a,b\right]\;=\;\left\{h\left(x\right):\;\left[a,b\right]\to R:\;{\left(\frac{\Psi (x)}{\Psi^{\left(1\right)}\left(x\right)}\frac{d}{dx}\right)}^{n-1}\;\left({\left(\Psi \left(x\right)\right)}^{\beta }\;h\left(x\right)\right)\;\in \;AC\left[a,b\right]\right\}$$

where AC[a,b] is the set of absolutely continuous functions on [a,b], which coincides with the space of primitives of Lebesgue measurable functions (see [1] (p. 3), and [4] (p. 2)). The condition f(x)∈AC[a,b] means that the function f(x) can be represented as

(18) $$f\left(x\right)\;=\;c\;+\;\int_{a}^{x}g\left(t\right)\;dt,\;\mathrm{where}\;\int_{a}^{b}\left|g\left(t\right)\right|\;dt<\infty ,$$

and *c* is a constant.

Theorem 4.7 of [68] gives sufficient conditions for the existence of the Hadamard-type fractional differential operator of a function with respect to another function.

**Theorem** **2.** *Let Ψ(x) be an increasing positive function on [a,b]⊂R, and $f\left(x\right)\in AC_{\delta^{\Psi },\beta }^{n}\left[a,b\right]$, α>0, n=[α]+1, β∈R.*

*Then, the Hadamard-type fractional differential operator $D_{\Psi ,a,x}^{\alpha ,\beta }\left[t\right]\;f\left(t\right)$ of f(x) with respect to Ψ(x) exists almost everywhere on [a,b].*

The proof of this theorem is given in [68].

Let us note the following special cases.

- For β=0, we obtain the Hadamard fractional operators with respect to Ψ(x).
- For β=0 and Ψ(x)=x, we obtain the Hadamard fractional integral and differential operators.
- For β=0 and $\Psi \left(x\right)=e^{x}$, we obtain the Riemann–Liouville and Caputo fractional integrals and derivatives.
- For β=0 and $\Psi \left(x\right)=e^{x^{\sigma }}$, we obtain the Erdelyi–Kober-type fractional integrals and derivatives (fractional operators with respect to $x^{\sigma }$.
- For β=0 and $\Psi \left(x\right)=e^{g(x)}$, we obtain the Riemann-Liouville and Caputo fractional operators of a function with respect to another function g(x).

In this paper, the Hadamard-type fractional integral and differential operators of a function with respect to another function are considered as the main object for entropy interpretations. The interpretation of HTFI and HTFD operators are special cases of the interpretation of the HIFI and HTFD operators of a function with respect to function Ψ(x)=x.

## 3. Entropy and Its Generalizations

Entropy is one of the basic concepts that play an important role in statistical mechanics and information theory [70]. The most widely used forms of entropy were proposed by Boltzmann and Gibbs for the statistical mechanics [71] and by Shannon for the information theory [72,73,74,75]. Later, certain generalized entropies such as the Renyi entropy [76] and the Tsallis entropy [77,78,79] were considered. The following two types of generalizations of entropy should also be noted. These are the so-called cumulative entropies and fractional entropies, which are discussed below.

### 3.1. Cumulative Entropies

As is well known, the standard approach to the description of information, which is related to a non-negative absolutely continuous random variable *X*, is based on the continuous entropy (differential entropy) of *X* defined by the equation

(19) $$H\left(X\right)\;=\;-\;\int_{0}^{\infty }\rho \left(t\right)\ln\rho \left(t\right)\;dt,$$

where ln(z) is the natural logarithm, and ρ(x) is the probability density function (PDF) of *X*. Entropy (19) can be represented as the mathematical expectation in the form

(20) H(X)=E[−lnρ],

where

(21) $$E\left[A\left(X\right)\right]\;=\;\int_{0}^{\infty }A\left(t\right)\;dF\left(t\right)\;=\;\int_{0}^{\infty }A\left(t\right)\;\rho \left(t\right)\;dt,$$

and F(t)=P(X≤t) is the cumulative distribution function

(22) $$F\left(x\right)\;=\;\int_{0}^{x}\rho \left(t\right)\;dt.$$

Cumulative entropy [80,81,82,83,84,85] is one of the modifications of the standard differential entropy (19). In works [80,81,82], the cumulative distribution function (CDF) of a random variable is proposed instead of probability density function (PDF). This approach gives an alternative measure of uncertainty that extends Shannon entropy to random variables with continuous distributions. This measure is called the cumulative residual entropy (CRE). In works [80,81,82], the cumulative residual entropy (CRE) is defined in the form

(23) $$E\left(X\right)\;=\;-\;\int_{0}^{\infty }P\left(X>t\right)\;\ln P\left(X>t\right)\;dt,$$

where P(X>t)=1−P(X≤t) is the probability that random variable *X* takes a value greater than *t*, and the cumulative distribution function (CDF) is F(t)=P(X≤t).

Let us note the main features of CR entropy in terms of its application from the point of view of authors of [80,81,82]:

Firstly, E(X) is more general than the Shannon entropy in that its definition is valid in the continuous and discrete domains.
Secondly, E(X) possesses more general mathematical properties than the Shannon entropy.
Thirdly, E(X) can be easily computed from sample data, and these computations asymptotically converge to the true values.
The properties of the cumulative residual entropy are given in works [80,81,82,83,85].

In paper [86] (see also [83,84]), the dynamic cumulative residual entropy was proposed. The CRE for the residual lifetime distribution with the survival function

(24) $${\bar{F}}_{x}\left(t\right)\;=\;\frac{\bar{F}\left(t\right)}{\bar{F}\left(x\right)},$$

where $\bar{F}\left(x\right)\;=\;1\;-\;F\left(x\right)$, and F(x)=P(X≤x) is defined by the equation

(25) $$E\left(X,t\right)\;=\;-\;\int_{x}^{\infty }{\bar{F}}_{x}\left(t\right)\;\ln{\bar{F}}_{x}\left(t\right)\;dt\;=\;\;-\;\int_{x}^{\infty }\frac{\bar{F}\left(t\right)}{\bar{F}\left(x\right)}\;\ln\left(\frac{\bar{F}\left(t\right)}{\bar{F}\left(x\right)}\right)\;dt.$$

Di Crescenzo and Longobardi [87] proposed and studied the cumulative entropy (CE), which is defined by the equation

(26) $$\mathrm{CE}\left(X\right)\;=\;-\;\int_{0}^{\infty }F\left(t\right)\;\ln F\left(t\right)\;dt,$$

and its dynamic version. The dynamic cumulative entropy is defined (see Equation (28) in [87]) in the form

(27) $$\mathrm{CE}\left(X,x\right)\;=\;-\;\int_{0}^{x}F_{x}\left(t\right)\;\ln F_{x}\left(t\right)\;dt\;=\;-\;\int_{0}^{x}\frac{F(t)}{F(x)}\;\ln\left(\frac{F(t)}{F(x)}\right)\;dt,$$

where F(t)=P(X≤t) is the cumulative distribution function (CDF), and

(28) $$F_{x}\left(t\right)\;=\;\frac{F(t)}{F(x)}.$$

Note that this entropy looks like the differential entropy (19). The main difference is that the probability P(X≤t) is used instead of P(X>t). This means that CE(X)≥0, which is not true for standard entropy (19).

Properties of the cumulative entropy in past lifetimes are available in [83,87,88]. In paper [89] (see also [90]), the authors propose a cumulative Tsallis entropy (CTE) measure and its dynamic version.

**Remark** **1.** *In order to be able to represent the cumulative entropies as some mathematical expectations, Equations (26) and (27) should be modified. To do this, the cumulative entropy can be defined by the equations*

(29) $$\mathrm{CE}\left(X\right)\;=\;-\;\int_{0}^{\infty }F\left(t\right)\;\ln F\left(t\right)\;dF\left(t\right).$$

*The dynamic cumulative entropy can be defined as*

(30) $$\mathrm{CE}\left(X,x\right)\;=\;-\;\int_{0}^{x}F_{x}\left(t\right)\;\ln F_{x}\left(t\right)\;dF_{x}\left(t\right),$$

*where $F_{x}\left(t\right)$ is defined by Equation (28), and*

(31) $$dF_{x}\left(t\right)\;=\;\frac{d}{dt}\left(\frac{F(t)}{F(x)}\right)\;dt.$$

*Using the standard mathematical expectation in the form*

(32) $$E\left[A\left(X\right)\right]\;=\;\int_{0}^{\infty }A\left(t\right)\;dF\left(t\right),$$

*Equation (29) can be represented as*

(33) CE(X)=−E[FlnF].

**Remark** **2.** *It should be noted that the cumulative entropies, which are defined by Equations (29) and (30), can be generalized to obtain two-parameter cumulative entropies in the form*

(34) $${\mathrm{CE}}^{\alpha ,\beta }\left(X\right)\;=\;\int_{0}^{\infty }{\left(F(t)\right)}^{\beta -1}\;{\left(-\;\ln F(t)\right)}^{\alpha -1}\;dF\left(t\right),$$

*where α>0 and β>0. The dynamic cumulative entropy can be defined as*

$${\mathrm{CE}}^{\alpha ,\beta }\left(X,x\right)\;=\;\int_{0}^{x}{\left(F_{x}\left(t\right)\right)}^{\beta -1}\;{\left(-\;\ln F_{x}\left(t\right)\right)}^{\alpha -1}\;dF_{x}\left(t\right)\;=\;$$

(35) $$\int_{0}^{x}{\left(\frac{F(t)}{F(x)}\right)}^{\beta -1}\;{\left(-\;\ln\left(\frac{F(t)}{F(x)}\right)\right)}^{\alpha -1}\;dF_{x}\left(t\right),$$

*where α>0 and β>0. Values (34) and (35) can be called the cumulative fractional entropies, which can be considered as a cumulative form of the fractional entropies. Some currently known fractional entropies are briefly described in the next subsection.*

### 3.2. Fractional Entropies

Using the fractional calculus, various generalizations of entropy are proposed (see review [91]). Let us note some extensions and fractional generalizations of the standard entropy.

In 2012 article [92], one-parameter fractional entropy was proposed in the form of the fractional integral equation

(36) $$S_{x}^{\alpha }\left(X\right)\;=\;-\;I_{0,x}^{\alpha }\left[t\right]\left(\rho (t)\;\ln\rho (t)\right)\;=\;\int_{0}^{x}\frac{{\left(x-t\right)}^{\alpha -1}}{\Gamma (\alpha )}\;\rho \left(t\right)\;\ln\rho \left(t\right)\;dt\;=\;\int_{0}^{x}\frac{{\left(x-t\right)}^{\alpha -1}}{\Gamma (\alpha )}\;\;\ln\rho \left(t\right)\;dF\left(t\right),$$

where the operator $I_{a,x}^{\alpha }\left[t\right]$ is the left-side Riemann–Liouville integral [4] (pp. 69–70).

Ubriaco in a 2009 paper [93] proposed the one-parameter fractional entropy. The continuous (differential) analogue of the Ubriaco fractional entropy can be written in the following form

(37) $$S_{U}\left(X\right)\;=\;\int_{0}^{\infty }\rho \left(t\right){\left(-\;\ln\rho \left(t\right)\right)}^{\alpha }\;dt\;=\;\int_{0}^{\infty }{\left(-\;\ln\rho \left(t\right)\right)}^{\alpha }\;dF\left(t\right),$$

where α∈(0,1]. The Ubriaco entropy is thermodynamically stable [93] and obeys the same properties as the Shannon entropy, with the exception of additivity. If α=1, Equation (37) gives standard entropy (19).

Wang in [94] proposed one-parameter fractional entropy in the context of the incomplete information theory. The continuous (differential) entropy analogue of the fractional Wang entropy can be written as

(38) $$S_{W}\left(X\right)\;=\;-\;\int_{0}^{\infty }{\left(\rho \left(t\right)\right)}^{\beta }\;\ln\rho \left(t\right)\;dt\;=\;-\;\int_{0}^{\infty }{\left(\rho \left(t\right)\right)}^{\beta -1}\;\ln\rho \left(t\right)\;dF\left(t\right).$$

The β-expectation ${\langle A\rangle }_{\beta }$ has been also considered

(39) $${\langle A\rangle }_{\beta }\;=\;\int_{0}^{\infty }A\left(t\right)\;{\left(\rho (t)\right)}^{\beta }\;dt,$$

which characterizes incomplete normalization ${\langle A\rangle }_{\beta }=1$.

Two-parameter fractional entropy was suggested in 2014 by Radhakrishnan, Chinnarasu, and Jambulingam [95]. A continuous (differential) entropy analogue of the RCJ fractional entropy can be written in the form

(40) $$S_{RCJ}\left(X\right)\;=\;\int_{0}^{\infty }{\left(\rho \left(t\right)\right)}^{\beta }\;{\left(-\;\ln\rho \left(t\right)\right)}^{\alpha }\;dt\;=\;\int_{0}^{\infty }{\left(\rho \left(t\right)\right)}^{\beta -1}\left(t\right)\;{\left(-\;\ln\rho \left(t\right)\right)}^{\alpha }\;dF\left(t\right),$$

where α∈(0,1], and β>0. For β=1, Equation (40) gives Equation (37). In the limit, when α=1, Equation (40) reduces to expression (38). For α=β=1, Equation (40) gives standard Equation (19).

## 4. Interpretation of Hadamard-Type Fractional Operators

### 4.1. Equation of HTFI Operator in Convenient Form

Equation (11), which defines the HTFI operator of function f(x) with respect to the function Ψ(x), can be rewritten in the form

(41) $$J_{\Psi ,a,x}^{\alpha ,\beta }\left[t\right]f\left(t\right)\;=\;\frac{1}{\Gamma (\alpha )}\int_{a}^{x}\;f\left(t\right)\;{\left(\frac{\Psi (t)}{\Psi (x)}\right)}^{\beta -1}{\left(-\;\ln\frac{\Psi (t)}{\Psi (x)}\right)}^{\alpha -1}\;{\left(\frac{\Psi (t)}{\Psi (x)}\right)}_{t}^{\left(1\right)}\;dt,$$

where x∈[a,b], and

(42) $${\left(\frac{\Psi (t)}{\Psi (x)}\right)}_{t}^{\left(1\right)}\;=\;\frac{d}{dt}\left(\frac{\Psi (t)}{\Psi (x)}\right).$$

Let us define the function

(43) $$F_{x}\left(t\right)\;=\;\frac{\Psi (t)}{\Psi (x)},$$

where a≤t≤x<b. Then, Equation (41) can be represented in the form

(44) $$J_{\Psi ,a,x}^{\alpha ,\beta }\left[t\right]f\left(t\right)\;=\;\frac{1}{\Gamma (\alpha )}\int_{a}^{x}\;f\left(t\right)\;{\left(F_{x}\left(t\right)\right)}^{\beta -1}{\left(-\;\ln F_{x}\left(t\right)\right)}^{\alpha -1}\;dF_{x}\left(t\right),$$

where x∈[a,b].

Equations (41) and (44) can be used to formulate possible interpretations of the Hadamard-type fractional integral and differential operators. Two types of interpretations are distinguished. The first interpretation intends to consider the function $F_{x}\left(t\right)$, which is defined by Equation (43), as a cumulative distribution function. In this interpretation, the basic representation of the HTFI operators is given by Equation (44). The second interpretation considers the function Ψ(t) as a cumulative distribution function. In this interpretation, the basic representation of the HTFI operators is described by Equation (41).

Let us write out separately the properties of the function Ψ(t), due to the definition of the fractional integral operator, and additional properties that allow us to consider the functions Ψ(t) or/and $F_{x}\left(t\right)$ as cumulative distribution functions.

### 4.2. Properties of Functions Ψ(x) and $F_{x}\left(t\right)$ used in HTFI Operator

In Definition 7, the following properties of the function Ψ(x) are used in [68]:

(1) Continuously differentiable function:
  (45) $$\Psi \left(t\right)\;\in \;C^{1}\left[a,b\right].$$
(2) Positive function:
  (46) Ψ(t)>0forallt∈[a,b].
(3) Increasing function:
  (47) $$\Psi^{\left(1\right)}\left(t\right)\;>\;0\;\mathrm{for}\;\mathrm{all}\;t\in \left[a,b\right].$$

Using the properties of the function Φ(x), for which HTFI is defined, one can describe properties of the function (43). Using properties of Ψ(x), one can prove that function (43) has the following properties:

(1) Continuously differentiable function:
  (48) $$F_{x}\left(t\right)\;\in \;C^{1}\left[a,b\right].$$
(2) Positive function:
  (49) $$F_{x}\left(t\right)\;>\;0,\;for\;\mathrm{all}\;t\in \left[a,b\right].$$
(3) Increasing function:
  (50) $$\frac{d}{dt}F_{x}\left(t\right)\;>\;0,\;for\;\mathrm{all}\;t\in \left[a,b\right].$$
(4) The limit on the left is equal to one:
  (51) $$\lim_{t\to x-}\;F_{x}\left(t\right)\;=\;1.$$

The above properties of the function Ψ(x) proposed in paper in [68] can be slightly weakened for our purposes.

Let (a,b), where −∞≤a<b≤+∞ is a finite or infinite interval of the real line R, and let α>0 and β∈R. Note that the function f(x) is assumed to be an integrable function such that $f\left(x\right)\in X_{\Psi ,q}^{p}\left(a,b\right)$, where 1≤p≤∞. Therefore, one can use β≥q. In addition, let Ψ(x) be an increasing and positive monotone function on (a,b], having a continuous derivative $\Psi^{\left(1\right)}\left(x\right)$ on (a,b), and $\Psi^{\left(1\right)}\left(x\right)\neq 0$ for a<x<b.

In addition, to use function (43) as the cumulative distribution function, we should consider only such functions Ψ(x) for which $F_{x}\left(t\right)$ is considered to be a cumulative distribution function.

### 4.3. Characteristic Properties of Cumulative Distribution Function

Let (R,B(R)) be the real line with the system B(R) of Borel sets. Let P=P(A) be a probability measure defined on the Borel subset *A* of the real line R. If A=(−∞,x], where x∈R, then

(52) F(x)=P(−∞,x]

is called the cumulative distribution function.

The following proposition describes basic properties of the cumulative distribution function.

**Proposition** **1.** *Let F(x) be a distribution function of a random variable X.*

*Then, F(x) satisfies the following properties:*

*I. Monotonicity: F(x) is a nondecreasing function. If $x_{1}<x_{2}$, then $F\left(x_{1}\right)\;\leq \;F\left(x_{2}\right)$.*

*II. Behavior at infinities*

(53) $$\lim_{x\to -\infty }\;F\left(x\right)\;=\;0,$$

(54) $$\lim_{x\to \infty }\;F\left(x\right)\;=\;1.$$

*III. Right-continuity: F(x) is a continuous function on the right and has a limit on the left at each x∈R.*

(55) $$\lim_{x\to x_{0}+}\;F\left(x\right)\;=\;F\left(x_{0}\right).$$

Proposition 1 is proved in [96] (p. 185), and in book [97] (p. 34).

One can formulate a theorem inverse to Proposition 1. This theorem shows what properties a function must have in order to be a cumulative distribution function.

**Theorem** **3.** *Let F=F(x) be a function on the real line R, which satisfies conditions I, II, and III. Then, there exists a unique probability (measurable) space (R,B(R),P) and a random variable X such that*

(56) P(X≤x)=F(x),

(57) P(a<X≤b)=P(a,b]=F(b)−F(a)

*for all a, b such that −∞≤a<b<∞.*

Theorem 3 is proved in book [97] (p. 35), as Theorem 3.2.1. Theorem 3 is also described in [96] (p. 185), as Theorem 1.

As a result, one can state that every function F(x) on the real line R, which satisfies conditions I, II, and III, is a cumulative distribution function.

### 4.4. Cumulative Fractional Entropy

Let Ψ=Ψ(x) be a function on the real line R, which satisfies conditions I, II, and III. Consider a cumulative distribution function Ψ(x), for which the conditions of the existence of the HTFI operator are satisfied.

Let (a,b), where −∞≤a<b≤+∞ is a finite or infinite interval of the real line R, and let α>0 and β∈R, such that β>q. In addition, let Ψ(x) be an increasing and positive monotone function on (a,b], having a continuous derivative $\Psi^{\left(1\right)}\left(x\right)$ on (a,b), and $\Psi^{\left(1\right)}\left(x\right)\neq 0$ for a<x<b. As a result, one can formulate the following definition.

**Definition** **9.** *Let (a,b), where −∞≤a<b≤+∞ is a finite or infinite interval of the real line R. Let Ψ(t) be a function, for which the following properties are satisfied:*

 *(1)*  *Continuously differentiable function:*
(58) $$\Psi \left(t\right)\;\in \;C^{1}\left(a,b\right).$$
 *(2)*  *Positive function:*
(59) Ψ(t)>0forallt∈(a,b).
 *(3)*  *Increasing function:*
(60) $$\Psi^{\left(1\right)}\left(t\right)\;>\;0\;for\;all\;\;t\in \left(a,b\right).$$
 *(4)*  *The limit on the right is zero:*
(61) $$\lim_{t\to a+}\;\Psi \left(t\right)\;=\;0.$$
 *(5)*  *The limit on the left is equal to one:*
(62) $$\lim_{t\to b-}\;\Psi \left(t\right)\;=\;1.$$

*The set of such cumulative distribution functions will be denoted by the symbol $C_{\Psi }^{1}\left(a,b\right)$.*

Let us give definitions of a cumulative fractional entropy and a dynamic cumulative fractional entropy, which are expressed through the HTFI operators.

**Definition** **10.** *Let (a,b), where −∞≤a<b≤+∞ is a finite or infinite interval of the real line R, and let α>0 and β∈R, such that β>q.*

*Let a cumulative distribution function F(x)=Ψ(x) belong to the set $C_{\Psi }^{1}\left(a,b\right)$, and let a function f(x) belong to the space $X_{\Psi ,q}^{p}\left(a,b\right)$, where 1≤p≤∞.*

*Then, there is a cumulative fractional entropy that is defined by equation*

(63) $$H_{F,a,b}^{\alpha ,\beta }\left[f\right]\;=\;\frac{1}{\Gamma (\alpha )}\int_{a}^{b}\;f\left(t\right)\;{\left(F(t)\right)}^{\beta -1}{\left(-\;\ln F(t)\right)}^{\alpha -1}\;dF\left(t\right).$$

*Then, there is a dynamic cumulative fractional entropy that is defined by equation*

(64) $$H_{F,a,x}^{\alpha ,\beta }\left[f\right]\;=\;J_{F,a,x}^{\alpha ,\beta }\left[t\right]\;f\left(t\right)\;=\;\frac{1}{\Gamma (\alpha )\;F(x)}\int_{a}^{x}\;f\left(t\right)\;{\left(\frac{F(t)}{F(x)}\right)}^{\beta -1}{\left(-\;\ln\frac{F(t)}{F(x)}\right)}^{\alpha -1}\;dF\left(t\right),$$

*where a<x<b, a≤t≤x.*

Using Theorem 1, one can obtain as a corollary the following proposition.

**Proposition** **2.** *Let (a,b), where −∞≤a<b≤+∞ is a finite or infinite interval of the real line R.*

*Let F(x)=Ψ(x) be a cumulative distribution function that belongs to the set $C_{\Psi }^{1}\left(a,b\right)$, and α>0, 1≤p≤∞, β∈R.*

*Then, cumulative fractional entropy (63) and dynamic cumulative fractional entropy (64) are bounded, if f(x) belongs to the space $X_{\Psi ,q}^{p}\left(a,b\right)$ and β≥q.*

The proposed cumulative fractional entropy and dynamic cumulative fractional entropy can be considered as a fractional generalizations of cumulative entropy (26) and dynamic cumulative entropy (27), which are proposed by Di Crescenzo and Longobardi in [87].

Note that for α=β=2 and f(x)=1 with a=0 and b=∞, Equation (63) gives the cumulative entropy (26), which are proposed by Di Crescenzo and Longobardi in [87], in which dt is replaced by dF(t).

Note that for α=β=2 and f(x)=1, Equation (64) with a=0 can be expressed through the dynamic cumulative entropy (27), which are proposed by Di Crescenzo and Longobardi in [87], in which dt is replaced by dF(t).

**Remark** **3.** *In Definition 10, the HTFI operators are used to proposed new types of entropy. Equations (63) and (64) contain a function f(x) in addition to the cumulative distribution function F(x) and the probability density function ρ(x) in the form dF(x)=ρ(x)dx. In order for Equations (63) and (64) to define generalizations of the concept of entropy, it is necessary to characterize the function f(x). As the simplest example, the unit function f(x)=1 can be considered as a function f(x). In this case, Equations (63) and (64) give generalizations of already known entropies.*

*In the general case, there are three following types of functions f(x).*

*(I) The case when f(x) is a function of the cumulative distribution function F(x), i.e., f(x)=f(F(x)). For example,*

(65) $$f\left(F\left(t\right)\right)\;=\;{\left(F(t)\right)}^{\gamma -1}\;{\left(-\;\ln F(t)\right)}^{\delta -1}.$$

*where γ>−α with α>0, and δ>−β> with β>q.*

*(II) The case when f(x) is a function of the cumulative distribution function F(x), i.e., f(x)=f(ρ(x)). For example,*

(66) $$f\left(\rho \left(t\right)\right)\;=\;{\left(\rho (t)\right)}^{\gamma -1}\;{\left(-\;\ln\rho (t)\right)}^{\delta -1}.$$

*where γ>−1 and δ≥0, or*

(67) $$f\left(\rho \left(t\right)\right)\;=\;-\;\ln_{q}\;\rho \left(t\right)\;=\;-\;\frac{{\left(\rho \left(t\right)\right)}^{q-1}-1}{q-1},$$

*where q≥0.*

*(III) The case when f(x) is a function of a function g(x) that can be considered as a functional parameter providing properties, the implementation of which allows Equations (63) and (64) to be interpreted as a generalization of the concept of entropy. As a simple example of such a function, one can consider constants, i.e., g(x)=λ. The function g(x) can the cumulative distribution function or the probability density function of another system.*

*One can consider combinations of these types of functions, that is, consider functions of the form f(t)=f(F(t),ρ(t),g(t)).*

*The dependence of the introduced generalizations of the concept of entropy on some function f(x) opens up additional possibilities and expands the list of possible characteristics of systems in statistical physics, information theory and quantum theory.*

**Remark** **4.** *Using cumulative distribution function (43), one can define cumulative fractional entropy in the form*

(68) $$H_{F,0,x}^{\alpha ,\beta }\left[f\right]\;=\;\frac{1}{\Gamma (\alpha )}\int_{0}^{x}\;f\left(t\right)\;{\left(F_{x}\left(t\right)\right)}^{\beta -1}{\left(-\;\ln F_{x}\left(t\right)\right)}^{\alpha -1}\;dF_{x}\left(t\right),$$

*where x∈[a,b], α>0 and β>0. Therefore, one can see that*

(69) $$H_{F,0,x}^{\alpha ,\beta }\left[f\right]\;=\;H_{F_{x},0,x}^{\alpha ,\beta }\left[f\right]$$

**Remark** **5.** *To simplify the notation, the entropy density function can be defined. Let us define the function*

(70) $$S_{F}^{\alpha ,\beta }\left(t\right)\;=\;{\left(F(t)\right)}^{\beta -1}\;{\left(-\;\ln F(t)\right)}^{\alpha -1},$$

*where α>0 and β>0. Note that function (70) can be interpreted as a fractional cumulative entropy density.*

*Using function (43), one also can define the function*

(71) $$S_{F}^{\alpha ,\beta }\left(t,x\right)\;=\;{\left(F_{x}\left(t\right)\right)}^{\beta -1}\;{\left(-\;\ln F_{x}\left(t\right)\right)}^{\alpha -1},$$

*where α>0 and β>0. Note that*

(72) $$S_{F}^{\alpha ,\beta }\left(t,b\right)\;=\;S_{F}^{\alpha ,\beta }\left(t\right),$$

*if $F\left(x\right)\in C_{\Psi }^{1}\left(a,b\right)$, i.e., F(b)=1.*

*For the special case α=β=1, function (70) has the form $S_{F}^{1,1}\left(t\right)\;=\;1$. For the special case α=β=2, function (70) has the form*

(73) $$S_{x}^{2,2}\left(t\right)\;=\;-\;F_{x}\left(t\right)\;\ln F_{x}\left(t\right).$$

*Equation (64) can be written as the mathematical expectation of the density of the cumulative entropy and function f(t) in the form*

(74) $$H_{F,a,b}^{\alpha ,\beta }\left[f\right]\;=\;E\left[f\left(t\right)\;S_{F}^{\alpha ,\beta }\left(t\right)\right].$$

*For the special case α=β=1, we have*

(75) $$H_{F,a,b}^{1,1}\left[f\right]\;=\;E\left[f(t)\right].$$

Let us note the following special cases. For β=0, the cumulative fractional entropies are described by the Hadamard fractional operators with respect to Ψ(x). For β=0 and Ψ(x)=x, the cumulative fractional entropies are represented by the Hadamard fractional integral and differential operators. In this case, the uniform distribution of the interval [0,1] can be considered.

**Definition** **11.** *Let (a,b), where −∞≤a<b≤+∞ is a finite or infinite interval of the real line R, and let α>0, n=[α]+1 and β∈R.*

*Let a cumulative distribution function F(x)=Ψ(x) belong to the set $C_{\Psi }^{1}\left(a,b\right)$.*

*Then, there is a dual cumulative fractional entropy that is defined by equation*

(76) $${\mathrm{DH}}_{F,a,b}^{\alpha ,\beta }\left[f\right]\;=\;H_{F,a,b}^{\alpha ,\beta }\left[D_{t}^{m,\beta }\;f\left(t\right)\right],$$

*where $H_{F,a,b}^{\alpha ,\beta }$ is defined in (63).*

*Then, there is a dual dynamic cumulative fractional entropy that is defined by equation*

(77) $${\mathrm{DH}}_{F,a,x}^{\alpha ,\beta }\left[f\right]\;=\;H_{F,a,x}^{\alpha ,\beta }\left[D_{t}^{m,\beta }\;f\left(t\right)\right],$$

*where a<x<b, a≤t≤x, $H_{F,a,x}^{\alpha ,\beta }$ is defined in (64).*

*Here, $D_{t}^{m,\beta }$ is the differential operator of integer order that is defined by Equation (4).*

Using Theorem 2, one can obtain as a corollary the following proposition.

**Proposition** **3.** *Let (a,b), where −∞≤a<b≤+∞ is a finite or infinite interval of the real line R.*

*Let α>0, n=[α]+1, β∈R, and F(x)=Ψ(x) be a cumulative distribution function that belongs to the set $C_{\Psi }^{1}\left(a,b\right)$.*

*Then, the dual cumulative fractional entropy (76) and the dual dynamic cumulative fractional entropy (76) exist almost everywhere on [a,b], if $f\left(x\right)\in AC_{\delta^{\Psi },\beta }^{n}\left[a,b\right]$.*

### 4.5. Examples

For an example of the dependence of fractional cumulative entropy on the function f(x), the following proposition and equations can be used.

**Proposition** **4.** *For ν>0, α>0 and β∈R, the following relations hold:*

(78) $$J_{\Psi ,c,x}^{\alpha ,\beta }\left[t\right]\left\{{\left(\Psi \left(t\right)\right)}^{-\beta }{\left(\ln\frac{\Psi (t)}{\Psi (c)}\right)}^{\nu -1}\right\}\;=\;\frac{\Gamma (\nu )}{\Gamma (\nu +\alpha )}{\left(\Psi \left(x\right)\right)}^{-\beta }{\left(\ln\frac{\Psi (x)}{\Psi (c)}\right)}^{\nu +\alpha -1},$$

(79) $$D_{\Psi ,a,x}^{\alpha ,\beta }\left[t\right]\left\{{\left(\Psi \left(t\right)\right)}^{-\beta }{\left(\ln\frac{\Psi (t)}{\Psi (c)}\right)}^{\nu -1}\right\}\;=\;\frac{\Gamma (\nu )}{\Gamma (\nu -\alpha )}{\left(\Psi \left(x\right)\right)}^{-\beta }{\left(\ln\frac{\Psi (x)}{\Psi (c)}\right)}^{\nu -\alpha -1}.$$

Proposition 4 is proved in work [68] as Proposition 3.13.

Using the Proposition 4, one can obtain the following property of the cumulative fractional entropy.

**Proposition** **5.** *Let (a,b), where −∞≤a<b≤+∞ is a finite or infinite interval of the real line R. Let F(t)=Ψ(t) be a cumulative distribution function, which belongs to the set $C_{\Psi }^{1}\left(a,b\right)$.*

*For a<c<b, ν>0, α>0 and β∈R, the dynamic cumulative fractional entropy is given by the equation*

(80) $$H_{F,c,x}^{\alpha ,\beta }\left[f\right]\;=\;\frac{\Gamma (\nu )}{\Gamma (\nu +\alpha )}{\left(F\left(x\right)\right)}^{-\beta }{\left(\ln\frac{F(x)}{F(c)}\right)}^{\nu +\alpha -1},$$

*if*

(81) $$f\left(t\right)\;=\;{\left(F\left(t\right)\right)}^{-\beta }{\left(\ln\frac{F(t)}{F(c)}\right)}^{\nu -1},$$

*where t>a*

For the case Ψ(t)=t, Equation (2).7.21 of Property 2.25 (see [4] (p. 113)) gives the equations

(82) $$J_{\Psi ,a,x}^{\alpha ,\beta }\left[t\right]\;t^{\nu }\;=\;{\left(\beta +\nu \right)}^{-\alpha }\;x^{\nu }.$$

(83) $$D_{\Psi ,a,x}^{\alpha ,\beta }\left[t\right]\;t^{\nu }\;=\;{\left(\beta +\nu \right)}^{\alpha }\;x^{\nu }.$$

**Proposition** **6.** *Let (a,b), where −∞<a<b<+∞ is a finite interval of the real line R.*

*Let F(t)=Ψ(t)=t/b be a cumulative distribution function of the uniform distribution on the interval [0,b], which belongs to the set $C_{\Psi }^{1}\left(a,b\right)$.*

*For ν>0, α>0 and β∈R, the dynamic cumulative fractional entropy is given by the equation*

(84) $$H_{F,0,x}^{\alpha ,\beta }\left[t^{\nu }\right]\;=\;{\left(\beta +\nu \right)}^{-\alpha }\;x^{\nu }.$$

Let us give some properties of the cumulative fractional entropy (76) and the dynamic cumulative fractional entropy (76).

(85) $$H_{F,a,b}^{\alpha ,\beta }\left[\lambda_{1}\;f_{1}\left(t\right)+\lambda_{2}\;f_{2}\left(t\right)\right]\;=\;\lambda_{1}\;H_{F,a,b}^{\alpha ,\beta }\left[f_{1}\left(t\right)\right]\;+\;\lambda_{2}\;H_{F,a,b}^{\alpha ,\beta }\left[f_{2}\left(t\right)\right],$$

(86) $$H_{F,a,b}^{\alpha ,\beta }\left[g\left(t\right)\;F\left(t\right)\right]\;=\;H_{F,a,b}^{\alpha ,\beta +1}\left[g\left(t\right)\right],$$

(87) $$H_{F,a,b}^{\alpha ,\beta }\left[g\left(t\right)\;\ln\;F\left(t\right)\right]\;=\;-\;H_{F,a,b}^{\alpha +1,\beta }\left[g\left(t\right)\right],$$

(88) $$H_{F,a,b}^{\alpha ,\beta }\left[g\left(t\right)\;F\left(t\right)\;\ln\;F\left(t\right)\right]\;=\;-\;H_{F,a,b}^{\alpha +1,\beta +1}\left[g\left(t\right)\right],$$

(89) $$H_{F,a,b}^{\alpha ,\beta }\left[g\left(t\right)\;{\left(F\left(t\right)\right)}^{\gamma }\;{\left(-\;\ln\;F\left(t\right)\right)}^{\delta }\right]\;=\;H_{F,a,b}^{\alpha +\gamma ,\beta +\delta }\left[g\left(t\right)\right],$$

where γ>−α with α>0, and δ>−β with β>q.

These properties can easily be proven using the determination of cumulative fractional entropy. Properties (86)–(88) are particular cases of property (89). Similar properties hold for the dynamic cumulative fractional entropy.

## 5. Conclusions

Interpretations of fractional integral and differential operators of non-integer order can play an important role for new applications of this tool in various fields of science. In this paper, an interpretation of Hadamard-type fractional integral and differential operators is proposed. It has been proved that such operators of integration and differentiation of one function with respect to another function can be considered as some type of cumulative fractional entropy. The concept of entropy plays an important role in information theory and statistical physics. The proposed interpretation opens up new possible applications of Hadamard-type fractional integral and differential operators in these sciences.

In this paper, only the interpretation of fractional operators is proposed. Important questions, which are not considered in this paper, are detailed studies of the properties of the proposed family of cumulative fractional entropies. In this case, the results obtained in the framework of the fractional Hadamard calculus can be used.

In conclusion, one can see that the proposed generalizations of the concept of entropy can be extended to the case of nonlocal probability theory by using general fractional probability density functions and general fractional cumulative distribution functions, which are proposed in [98,99].

## Data Availability

Not applicable.
